# Congenital Diaphragmatic Hernia With Kidney and Spleen Herniation in the United Arab Emirates: A Case Report

**DOI:** 10.7759/cureus.26732

**Published:** 2022-07-11

**Authors:** Anas Zahid, Faisal A Nawaz, Ruthwik Duvuru, Yousef S Alabrach, Aftab Ahmed

**Affiliations:** 1 Medicine, Mohammed Bin Rashid University of Medicine and Health Sciences, Dubai, ARE; 2 Medicine, Sheikh Khalifa Medical City, Abu Dhabi, ARE; 3 Pediatric Surgery, Mediclinic Welcare Hospital, Dubai, ARE

**Keywords:** middle-east, kidney herniation, intrathoracic kidney, laparotomy, hypoplastic lung, bochdaleck hernia, newborn, respiratory distress, congenital diaphragmatic hernia

## Abstract

Congenital diaphragmatic hernia (CDH) is a severe congenital anomaly that leads to herniation of abdominal viscera to the chest, which presents with respiratory distress shortly after birth. Spleen herniation is a rare finding, and kidney herniation is even more exceedingly rare. We hereby report a case of a neonate that developed severe respiratory distress secondary to CDH. After confirming the diagnosis with chest and abdominal X-ray and initial stabilization, the patient underwent laparotomy, which revealed a large diaphragmatic defect with herniation of the ileum, colon, spleen, and left kidney. Contents were reduced to the abdomen, and the defect was repaired. The patient had a complete recovery with no complications. After reviewing the literature, we noticed the paucity of data in the Middle East region regarding the disease burden and the increased rate of complications with delayed diagnosis. Therefore, we believe that this case, which was presented in the United Arab Emirates with kidney and spleen herniation and received prompt management, is a valuable addition to the literature.

## Introduction

Congenital diaphragmatic hernia (CDH) is a developmental anomaly associated with herniation of abdominal viscera into the thoracic cavity. Affected individuals usually present with respiratory distress in the first few hours of life. The incidence of CDH ranges between 0.8-5/10,000 births and varies across populations [1,2]. A 2014 study reported the prevalence of CDH in Europe to be 2.3%, with males affected more than females at a ratio of 10:7 [3].

Posterolateral hernias, also known as Bochdalek hernias, are the most common type accounting for about three-fourths of cases, with the majority occurring on the left side [4]. Delayed diagnosis can contribute to significant morbidities, such as intestinal strangulation [5]. Kidney herniation is an exceedingly rare finding; the prevalence was reported to be less than 0.25% [6].

We present a case of CDH that presented in the first few hours of life in the United Arab Emirates (UAE). The lack of available research on the pathophysiology of CDH and disease burden in the Middle East region, alongside the increased rate of complications with delayed diagnosis, and the presence of kidney and spleen herniation, makes our case an important addition to the literature.

## Case presentation

We report the case of a male patient who was born at 38 weeks, by vaginal delivery, to a mother who was G1P0, with A positive blood group, and normal antenatal scans. The patient had a birth weight of 2,695 g, with an Apgar score of 8 in the first minute and 9 in the fifth minute of life. The patient cried immediately after initial stimulation and was admitted to the postnatal ward.

**Figure 1:**
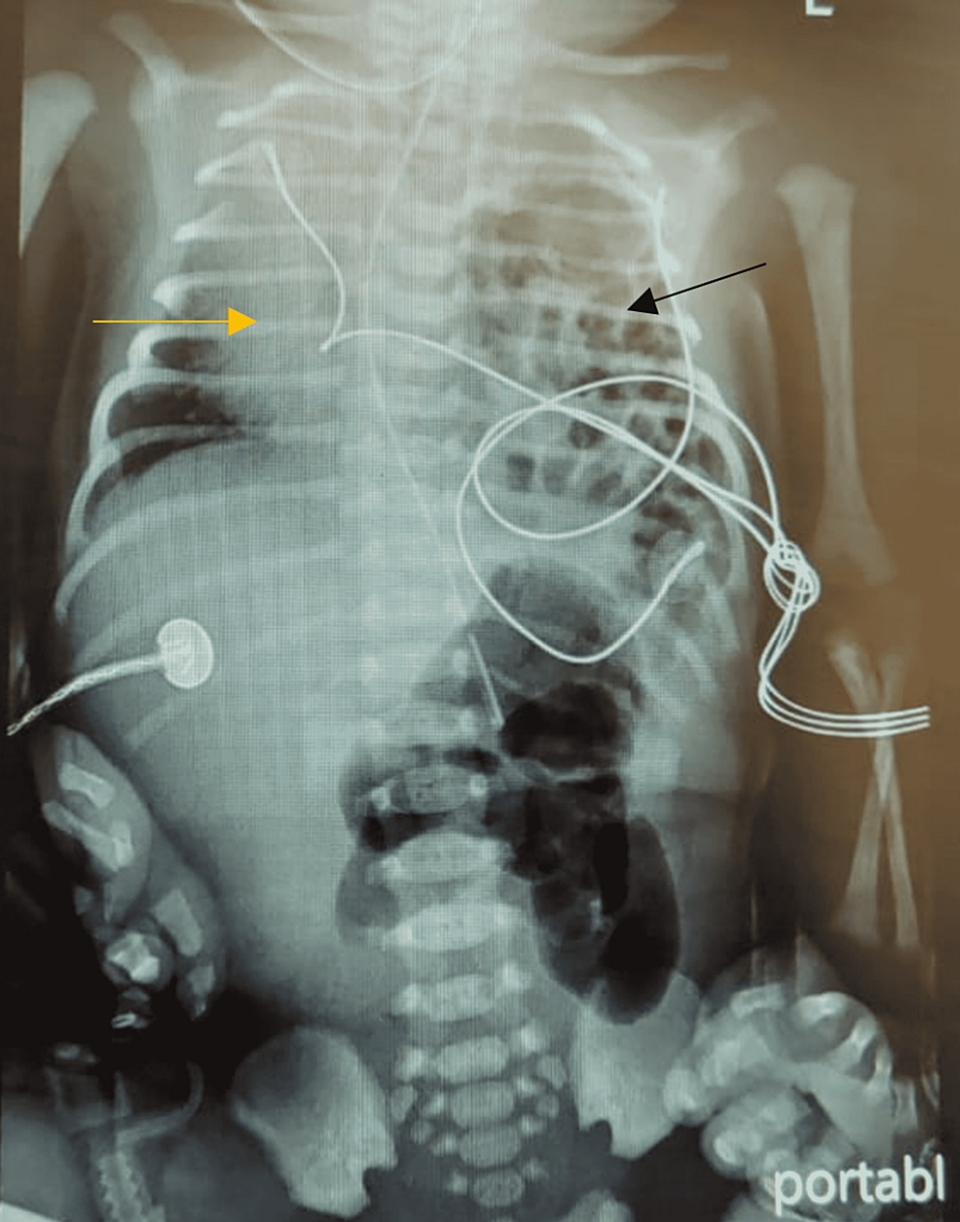
X-ray film showing multiple air-filled structures occupying the left hemithorax (black arrow). The mediastinum is shifted to the right (yellow arrow).

At 11 hours of life, the patient developed severe respiratory distress, which involved grunting, subcostal recession, tachypnea, and a SpO_2_ of 79%-81%. He was immediately shifted to the neonatal intensive care unit (NICU) to evaluate his severe respiratory distress. Capillary blood gas (CBG) showed metabolic acidosis (ph 7.04, PCO_2_ 89.1, HCO_3_ 15.7). Following this finding, the patient was started initially on a high flow nasal cannula (HFNC) 6L/min. The chest x-ray showed multiple air locules with well-defined walls in the left hemithorax and a paucity of bowel gas in the abdomen. The mediastinum was shifted to the right, and there was a large area of collapse in the right lung. Figure 1 demonstrates the x-ray findings. These findings were in line with a large left-sided diaphragmatic hernia.

After confirmation of a left-sided diaphragmatic hernia through an x-ray of the chest and abdomen, the patient was intubated following premedication with atracurium and morphine and started with mechanical ventilation (PC/AC mode, p24/5, R 60/min, FiO_2_ 100%). Umbilical artery catheter (UAC) and peripherally inserted central catheter (PICC) lines were inserted aseptically. Blood for the partial septic screen was sent. The patient was kept null per oral (NPO) and was started on Intravenous fluids dextrose 10% 60mL/kg/day. IV antibiotics were started. Blood sugar and gases were monitored. An echo was also performed, which showed the heart shifted to the right with normal anatomy and function. On ultrasound, bowel loops were seen in the left hemithorax. It also showed the absence of the left kidney from the renal bed. The main differentials for this finding were agenesis or ectopia. The patient was scheduled for surgery the next day to repair the congenital defect.

**Figure 2:**
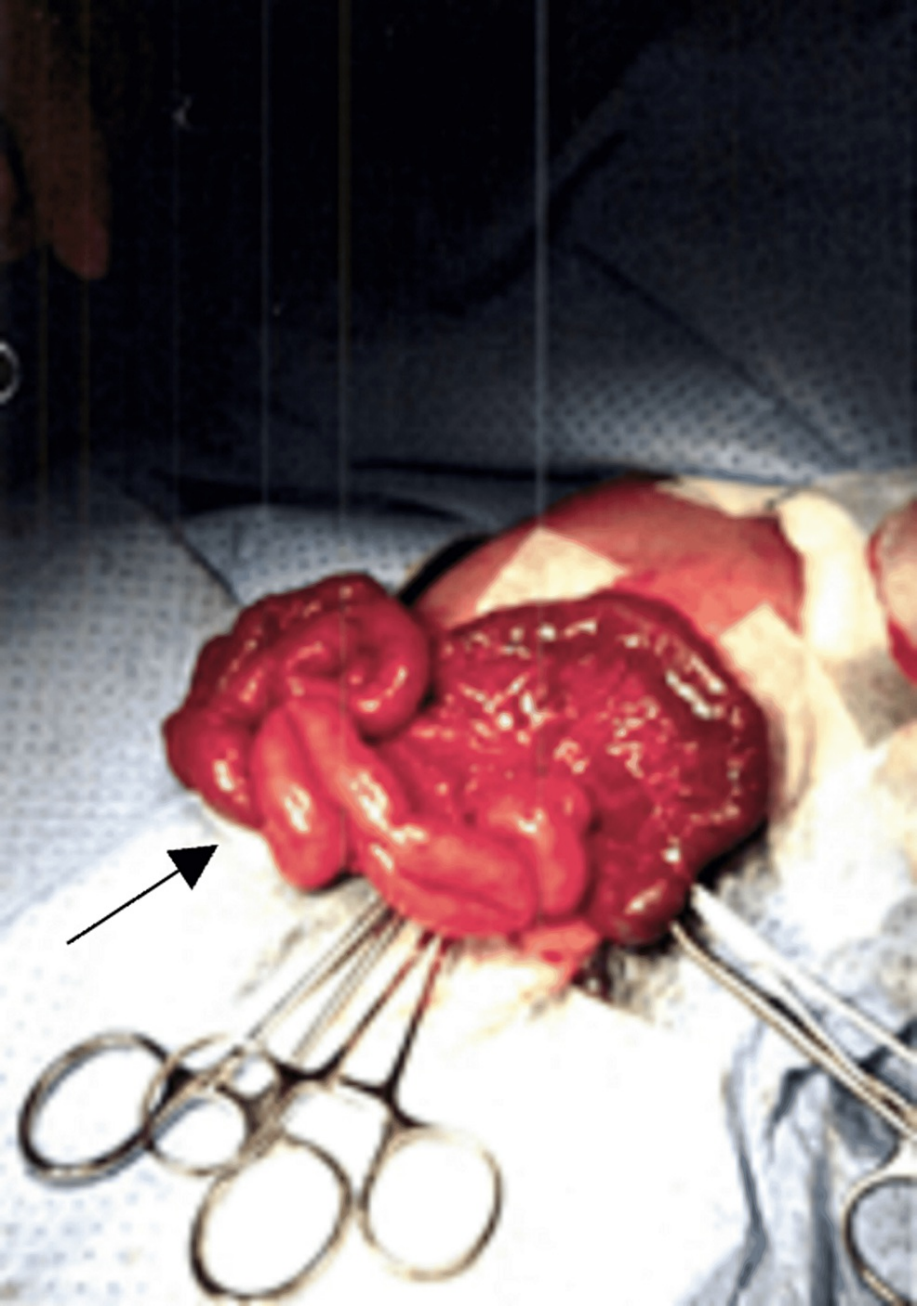
Intraoperative image of the herniated contents (arrow).

The patient underwent exploratory laparotomy, which revealed a large defect in the left part of the diaphragm with herniation of the left kidney, spleen, ileum, jejunum, and colon. The contents of the herniation were reduced back into the abdomen. Figure 2 shows an intraoperative image of the herniated contents that were reduced back into their normal position. Hypo-plastic lung was seen in the thoracic cavity. The bowel was checked for malrotation, which was ruled out. There was no raised intra-abdominal pressure, and the wound was closed with interrupted sutures. Hemostasis was maintained throughout. The patient had an uneventful recovery without complications and normal follow-up visits.

## Discussion

Abdominal organs such as the bowel, spleen, and kidney can travel to the thoracic cavity through a defect in the diaphragm. This can compress the lungs and shift the mediastinum to the opposite side [7]. Contingent on the amount of pulmonary compression, there may be a discernible reduction of the bronchioles, restricted development of alveoli, and muscular hypertrophy of the arterioles leading to bilateral pulmonary hypoplasia with a more significant impact on the ipsilateral lung. Patients born with this condition are more likely to develop persistent pulmonary hypertension, retaining the fetal circulation to the lungs. The right to left shunt leads to acidosis, respiratory distress, and pulmonary vasoconstriction. This can cause reduced lung compliance [8]. These mechanisms most likely explain the symptoms present in our patient, which includes hypoplastic lung along with respiratory distress and acidosis.

Several studies in the review indicated maternal education, maternal smoking, alcohol use during pregnancy, maternal obesity, multiple pregnancies, maternal medical conditions, such as diabetes and hypertension, and paternal factors, such as age and occupation, as potential risk factors. A cumulative 600 cases out of 5,927 cases in 30 studies had underlying genetic etiology, with the most common anomaly being trisomy 18 [3]. No specific risk factor was identified in this patient.

The embryological physiology behind CDH was initially thought to be due to the absence of fusion between various diaphragm parts resulting in a pleuroperitoneal canal [9]. However, this remains controversial. Prenatal diagnosis by ultrasound detects around 50% of CDH cases [10]. However, in our case, the patient had normal prenatal scans. Interestingly, patients diagnosed prenatally are considered a higher risk group than those diagnosed postnatally, likely reflecting that severe lesions will be more apparent in prenatal ultrasound [11].

Right-sided CDH is relatively difficult to diagnose compared to left-sided CDH. Several fetal predictors of outcomes have been reported, i.e., associated anomalies, the extent of lung hypoplasia, position of the liver, and contralateral lung size to head circumference [10]. Liver herniation has been associated with a worse prognosis. Our patient had no associated anomalies, and the liver was in a normal position. This might have contributed to the favorable outcome of the case.

The defect in the diaphragm allows both the small and large intestinal loops to herniate into the thoracic cavity along with other solid abdominal viscera [12]. A splenic herniation is rare, which was present in our case. One exceedingly rare occurrence that was also present is the herniation of the kidney. According to the literature, intrathoracic renal ectopia (IRE) has less than 0.01% occurrence [6]. It can occur as an isolated anomaly, or it can be associated with CDH, which has a 0.25% prevalence [6].

IRE associated with CDH is more common on the left side, possibly due to the liver position on the right side, and it is more common in males [13]. IRE can be a result of herniation through the diaphragmatic defect, or it can represent true embryological ectopia, which maybe is secondary to accelerated ascent or delayed closure of the pleuroperitoneal membrane [13]. One possible method to differentiate the two is to compare prenatal and postnatal ultrasounds; in our case, the prenatal US showed the normal location of the kidneys, but postnatally the US showed an absence of left kidney from the left renal bed. This indicates herniation rather than true ectopia [6]. Moreover, embryological IRE is associated with other anatomical renal anomalies, such as elongated ureter [13]. This was not present in our case.

The type of respiratory distress described in our patient may advance to even more severe distress and respiratory failure if not managed promptly. The physical examination can reveal displaced heart sounds, an excavated abdomen, and bowel sounds in the chest. On imaging, we saw a hemithorax filled with multiple air locules with well-defined walls, which reflects bowel loops and abdomen with a paucity of gas, which are well-characterized findings of the disease [14]. In our case, the disease was quickly identified by accurate interpretation of radiographs, and management was appropriately started. No significant morbidities have occurred, emphasizing the importance of a timely diagnosis.

Treatment involves providing timely oxygenation to patients with severe respiratory distress. To prevent pulmonary hypertension, the patient should be intubated straight away to achieve rapid chest decompression with abdominal straps once the diaphragmatic hernia has been confirmed by imaging. A nasogastric (NG) tube should be placed as well. Special care should be implemented when using assisted ventilation to prevent injury to the opposite lung by keeping a low inspiratory pressure. The surgical repair of the defect should only be scheduled once the patient has stabilized. In the 1980s, emergency surgery to remove abdominal viscera from the thoracic cavity was the go-to management plan. however, that only led to a worse prognosis due to the subsequent worsening of pulmonary hypertension [15].

Therefore, delayed surgery coupled with gentle ventilation is currently the management modality. Currently, surgical repair should only be done after cardio-respiratory stability has been achieved. It is unclear how long surgery should be delayed; however, a few days to a few weeks may be beneficial in conjunction with extra-corporeal membrane oxygenation (ECMO) [16]. In our case, the surgery was delayed to the next day after stabilizing the patient, which aligns with the aforementioned management. The patient responded well, supporting the importance of early surgical correction but only after initial stabilization. The surgery usually involves subcostal laparotomy with the placement of abdominal contents back into their position. Following this, the defect is permanently closed. Pulmonary hypertension and hypoplasia persist to be a long-term mortality risk for these patients [17].

We believe that our case report contributes to offering neonatologists, pediatric surgeons, and maternal-fetal medicine specialists with tangible prognostic data and outcomes for counseling families regarding this condition. Moreover, our case involves splenic and kidney herniation through the diaphragmatic defect, which is rare, and very few cases have been reported in the literature. Most importantly, Previous research showed that patients with CDH in the Middle East have unique characteristics compared to the available literature and suggests the importance of having a CHD registry specific to this patient population [18]. Therefore, this case adds to the available literature on the Middle East population in general and the UAE in particular, which can guide further similar studies.

## Conclusions

This case report describes a rare clinical presentation of a newborn's symptomatic CDH associated with the herniated spleen and left kidney. The diaphragmatic defect allowed a substantial amount of contents to herniate into the thoracic cavity. This congenital condition is fatal if not timely treated; therefore, understanding safe surgical methods is vital. Moreover, this case is one of few cases reported in the Middle East, particularly in the UAE, which might assist management decisions in this unique population.
